# A Diverse Membrane Interaction Network for Plant Multivesicular Bodies: Roles in Proteins Vacuolar Delivery and Unconventional Secretion

**DOI:** 10.3389/fpls.2020.00425

**Published:** 2020-04-30

**Authors:** Shuai Hu, Yan Li, Jinbo Shen

**Affiliations:** State Key Laboratory of Subtropical Silviculture, Zhejiang A & F University, Hangzhou, China

**Keywords:** PVC/MVBs, TGN, PM, membrane interaction, vacuolar trafficking, autophagosome, protein secretion

## Abstract

Vesicle trafficking between the membrane-bound organelles in plant cells plays crucial roles in the precise transportation of various materials, and thus supports cell proliferation and cellular polarization. Conventionally, plant prevacuolar compartments (PVCs), identified as multivesicular bodies (MVBs), play important roles in both the secretory pathway as intermediate compartments and the endocytic pathway as late endosomes. In recent years, the PVC/MVBs have been proposed to play important roles in both protein vacuolar delivery and unconventional secretion, but several important questions on the new regulators and environmental cues that coordinate the PVC/MVB–organelle membrane interactions and their biological significances remain. In this review, we first summarize the identity and nature of the plant PVC/MVBs, and then we present an update on our current understanding on the interaction of PVC/MVBs with other organelles in the plant endomembrane system with focus on the vacuole, autophagosome, and plasma membrane (PM) in plant development and stress responses. Finally, we raise some open questions and present future perspectives in the study of PVC/MVB–organelle interactions and associated biological functions.

## Introduction

**FIGURE 1:**
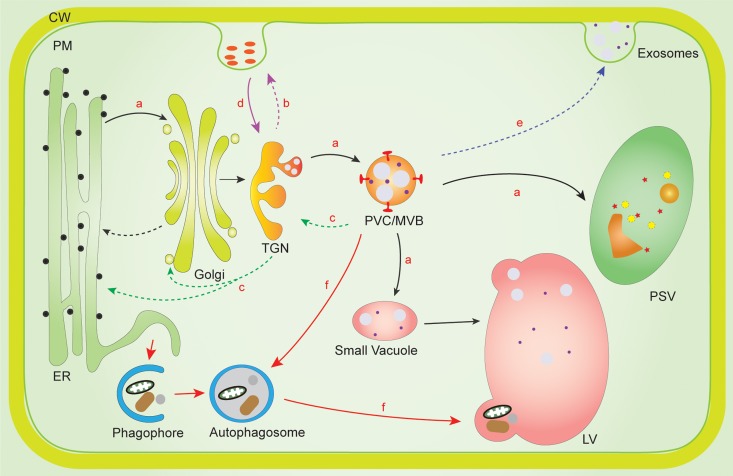
Membrane interaction network of PVC/MVBs with other organelles in the plant endomembrane system. **(a,b)** In the plant secretory pathway, proteins destined for LV/PSV are sorted from TGN en route to PVC/MVB, and later deposited into LV/PSV (route **a**, black solid arrow). Some proteins without vacuolar sorting signal will be secreted outside of the cell from the TGN via the “default pathway” (route **b**, purple dashed arrow). **(c)** Proteins can be recycled from either MVB/PVC, TGN, or Golgi as retrograde protein transport (route **c**, green dashed arrow). **(d)** In the endocytic pathway (route **d**, purple solid arrow), proteins are internalized from the plasma membrane or extracellular space and first reach the Early Endosomes/TGN. From there, they either move to Late Endosome/PVC/MVB for further transport to the LV for degradation or are recycled back from TGN to the PM. **(e)** Protein secretion can be mediated by PVC/MVB-mediated unconventional protein secretion routes (route **e**, blue dashed arrow). **(f)** In autophagy pathway, PVC/MVBs can interact with autophagosome for materials delivered into the LV for degradation (route **f**, red solid arrow). ER, endoplasmic reticulum; CW, cell wall; LV, lytic vacuole; PM, plasma membrane; PSV, protein storage vacuole; PVC/MVB, prevacuolar compartment/multivesicular bodies; TGN, *trans* Golgi network.

All eukaryotic cells have a functionally interrelated endomembrane system, which is “connected” by types of vesicle-mediated movement of materials. The plant endomembrane system consists of numerous conserved membrane-bound organelles, including the nuclear envelope, the endoplasmic reticulum (ER), the Golgi apparatus, the *trans*-Golgi network or early endosome (TGN/EE), the prevacuolar compartment/multivesicular body or late endosome (PVC/MVB/LE), and the vacuole. Each organelle membrane consists of a unique complex mixture of phospholipids and proteins, which help them perform specific functions in vesicle fission and fusion during protein transport. Over the past decades, numerous unique organelles and multiple protein transport pathways in the plant endomembrane system have been identified and characterized, especially the plant secretory pathway and endocytic pathway (Figure 1; Viotti et al., 2010; Rabouille, 2017).

In the plant secretory pathway (Figure 1, route a, black solid arrow), newly synthesized soluble proteins contain a signal peptide to ensure translocation into the ER lumen for correct folding. Subsequently, they are transported into Golgi apparatus followed by the TGN. Proteins with vacuolar sorting signals are recognized by vacuolar sorting receptors (VSRs) as cargo at TGN and are then transported to PVC/MVBs, which contain numerous intraluminal vesicles (ILVs) (Tse et al., 2004), whereas the proteins without vacuolar sorting signals will be secreted to extracellular space (Figure 1, route b, purple dashed arrow; Shen et al., 2013). Then, the VSRs dissociate from cargo and are recycled back to the TGN for another round of cargo sorting (Figure 1, route c, green dashed arrow). Finally, the cargo proteins presented in the PVC/MVBs are transported into lytic vacuole (LV) or protein storage vacuole (PSV) after the fusion of the PVC/MVBs with the vacuole. This is the traditional model for protein transport to the plant vacuole. Against this, a new emerging model for VSR-cargo proteins sorting and receptor recycling has emerged, which suggests that the VSR-cargo sorting process may be initiated already at the ER or the *cis*-Golgi, and then cargoes are released from the receptors in the TGN from where the VSRs are transported back through the retromer complex for another round of sorting (Niemes et al., 2010a, b; Scheuring et al., 2011; Künzl et al., 2016; Robinson and Neuhaus, 2016; Früholz et al., 2018; Salanenka et al., 2018).

In the endocytic pathway (Figure 1, route d, purple solid arrow), materials are internalized at the PM by invagination and budding of limited subdomains of membrane into endocytic vesicles and are targeted to the TGN, which is also defined as EE in plants (Lam et al., 2007). After that, a subdomain of TGN/EE membrane functions as a recycling endosome that mediates the return of receptor proteins or lipids back to the PM for another round of sorting, while other subdomains of the TGN/EE membrane mature into LEs, which are identical to the PVC/MVBs in plant cells (Tse et al., 2004). The matured LEs or PVC/MVBs finally fuse with vacuole. Thus, proteins or cargo from both secretory and endocytic pathways converge on the TGN/EE and then are transported to PVC/MVBs by different sorting machineries.

Recently, significant progress has been achieved on the identity, biogenesis, membrane interactions, and biological functions of PVC/MVBs in plant cells. In this review, we give an update summary on the identification and biogenesis of plant PVC/MVBs and then discuss its membrane fusion with vacuole as well as the crosstalk with autophagosomes in regulating vacuolar degradation pathways. In addition, we highlight the membrane interaction between PVC/MVBs and the PM for its critical function in plant pathogen defense. Last, we give a brief comment on open questions and perspectives for future studies on plant PVC/MVBs.

## What Are Plant Prevacuolar MVBS?

In mammals, endocytosed proteins from the PM are first delivered to EEs and then sent to LEs before final delivery to lysosomes. LEs and EEs are two distinct organelles and have different luminal pHs and different proteins attached to their membranes (Russell et al., 2006). LEs were first noted in mammalian cell by electron microscopy in Palade (1955). Generally, the diameter of LEs is 100–600 nm with the ILVs inside up to 50 nm in diameter. Because LEs contain numerous ILVs inside, the LEs are also called MVBs. On the basis of precedents from mammalian and yeast cells, LEs mainly function as an intermediate organelle between the TGN and lysosomes/vacuole for protein transport. Thus, LEs are also sometimes called prelysosomal compartments (PLCs) or prevacuolar compartments (PVCs) (Jahraus et al., 1994; Luzio et al., 2003; Russell et al., 2006). In the secretory pathway of mammals, lysosomal protein transported from EE/TGN to LE is mediated by mannosyl 6-phosphate receptors (MPRs), which recruit lysosomal acid hydrolases at the TGN. As the TGN matures into the LE, the hydrolases are released from the MPRs owing to the acidic environment, and MPRs are then recycled back to the TGN for another round of sorting (Chen et al., 1997). A similar sorting mechanism is also found in yeast, where the sorting receptor Vps10p recycles between the Golgi apparatus and the PVCs for the vacuolar sorting of carboxypeptidase Y (CPY) (Conibear and Stevens, 1998).

Following the research works in mammals and yeast, it is thus possible to use vacuolar sorting receptor (VSR), which recycles between the Golgi apparatus and vacuole as a protein marker to define the plant PVCs. Indeed, the pea BP-80 protein, the first identified VSR in plants functioning in sorting acid hydrolases to the vacuole, was found in both dilated ends of Golgi cisternae and a morphologically undefined “prevacuole” structure in pea root tip cells (Paris et al., 1997). Subsequent study using Arabidopsis VSR homolog AtELP/AtVSR1 suggests that the VSR can be found in both the uncharacterized reticulotubular compartments and the 100-nm electron-dense uncoated vesicles (later named as PVC/MVBs) in root tip cells of Arabidopsis (Ahmed et al., 1997; Sanderfoot et al., 1998). In addition, VSR antibodies were labeled and concentrated at MVBs in the thin sections from high-pressure frozen/freeze-substituted samples or purified MVBs from the BY-2 cell line, respectively (Tse et al., 2004). Moreover, VSR antibody-labeled MVBs have also been illustrated in both developing Arabidopsis seeds and germinated mung bean (Hara-Nishimura et al., 1998; Tse et al., 2004; Wang et al., 2007). Thus, the PVCs in plants are identified as the MVBs and the VSR antibodies can be used as a PVC/MVB marker.

The plant VSR is a type I membrane protein, which behaves as one transmembrane domain (TMD) with its N-terminus (NT) facing the lumen and its C-terminus (CT) facing the cytoplasm. The NT of VSR is responsible for cargo binding, while the TMD and CT regions are essential and sufficient for PVC/MVB targeting of VSR in plant cells (Li et al., 2002). The artificial reporter proteins containing the TMD and CT regions of VSRs (e.g., GFP-BP-80TMD/CT) colocalize with VSR antibody-labeled punctate dots, and thus are sufficient to be used as protein markers of the PVC/MVBs in Arabidopsis (Sohn et al., 2003; Lee et al., 2004; Miao et al., 2006; Zhu et al., 2019).

**FIGURE 2:**
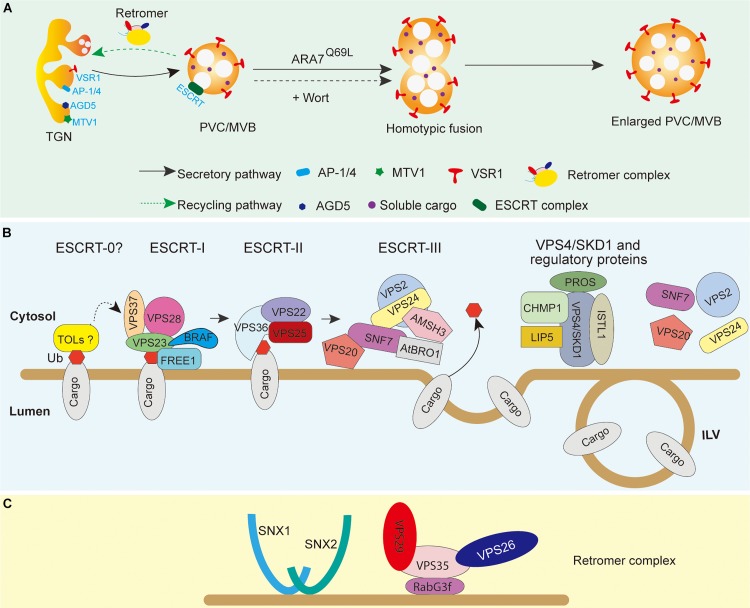
PVC/MVB biogenesis and maturation in plant cells. **(A)** PVC/MVBs mature from the tubular–vesicular TGN. Overexpression of ARA7^Q69L^ and wortmannin treatment can induce enlargement of PVC/MVBs. The PVC/MVB membrane localization of VSRs can be used as a marker of normal or enlarged PVC/MVBs. **(B)** The working model of ESCRTs in ILV formation and membrane protein endosome sorting. Ubiquitinated cargoes are recognized by the ESCRT-0-like protein TOLs and then transferred to ESCRT-I and ESCRT-II by the ubiquitin-binding proteins FREE1, VPS23, and VPS36. ESCRT-III is then activated by ESCRT-II via the interaction between VPS25 and VPS20 to constrict and invaginate the membrane of PVC/MVBs to form ILVs. The ubiquitin of cargoes can then be removed by the deubiquitinating enzyme AMSH3, which is recruited by AtBRO1 in the ESCRT-III complex. At last, the VPS4/SKD1 complex is recruited by ESCRT-III accessory proteins to dissociate the ESCRT-III complex from the PVC/MVB membrane. **(C)** Tentative model of the assembly of the core retromer to endosomal membranes in plants. The retromer complex is composed of a dimer of SNXs and of the core retromer consisting of VPS26, VPS29, and VPS35. The core retromer is recruited to the endosomal membrane by the Rab7 GTPase RABG3f. Membrane proteins (e.g., VSRs and PINs) can be recycled by the attachment of the retromer complex to the membrane. AtBRO1, Arabidopsis BRO1-like domain containing protein 1; AMSH3, associated molecular with SH3 domain of STAM 3; BRAF, bro1-domain protein as FREE1 suppressor; CHMP, charged multivesicular body protein; ESCRT, endosomal sorting complexes required for transport; FREE1, FYVE domain protein required for endosomal sorting 1; LIP5, protein homolog of mammalian lyst-interacting protein 5; VSR, vacuolar sorting receptor; PIN, PIN auxin efflux carriers; PROS, positive regulator of SKD1; SKD1, suppressor of k^+^ transport growth defect 1; SNF7, sucrose non-fermenting 7; SNX, sorting nexins; TOL, TOM1-like; ISTL1, increased salt tolerance 1; Wort, wortmannin; ILV, intraluminal vesicles; VPS, vacuolar protein sorting.

The yeast PEP12p is a member of Qa-SNARE proteins and functions in the vacuolar proteins transport (von Mollard et al., 1997; Gerrard et al., 2000). In Arabidopsis, AtPEP12 localizes between the Golgi and the vacuole as small circular membrane-bound structures—PVCs (da Silva Conceição et al., 1997). Besides, Rab5 family GTPases RHA1 and ARA7 are also reported to colocalize with AtPEP12. Thus, RHA1 and ARA7 are also used as a marker of the PVC/MVBs (Kotzer et al., 2004; Lee et al., 2004). Moreover, overexpression of the constitutively active GTP-bound mutant of ARA7, GFP-ARA7^Q69L^, can induce the enlarged PVC/MVB formation, present as ring-like structures under the confocal microscope because of their homotypic fusion. Moreover, overexpression of the constitutively active GTP-bound mutant of ARA7, GFP-ARA7^Q69L^, can induce the enlarged PVC/MVB formation, present as ring-like structures under the confocal microscope, which properly originate from their homotypic membrane fusion (Jia et al., 2013). Similarly, the dilated ring-like structures of the PVC/MVBs are also labeled by fluorescent-tagged VSR or ARA7 upon treatment by wortmannin, an inhibitor of phosphatidylinositol-3 kinase (PI-3 kinase and Vps34p in yeast) (Corvera et al., 1999; Tse et al., 2004). Such wortmannin-induced enlargement of MVBs have been supposed to be formed by the fusions between the TGN and PVC/MVBs, as well as the homotypic fusions of the PVC/MVB membrane (Wang et al., 2007; Wang J. et al., 2009). Currently, the overexpressed ARA7^Q69L^ and wortmannin-induced enlargement of MVBs have been used as specific tools to identify the plant PVC/MVBs (Figure 2A).

## PVC/MVB Biogenesis and Maturation

In mammalian cells, EE has typically two structurally distinct domains: a central nearly spherical structure with ILVs inside and an extensive tubular network with clathrin-coated buds projecting into the cytoplasm (Griffiths and Gruenberg, 1991; Tooze and Hollinshead, 1991). The TGN in plants shares many typical features with the EE in mammalian cells on the morphological structure (Stoorvogel et al., 1996; Kang and Staehelin, 2008; Toyooka et al., 2009) and functions (Lam et al., 2007; Cai et al., 2012). Indeed, the current study demonstrates that the TGN in plant cell displays tubular–vesicular structures with several coated buds, which functions for anterograde traffic to the vacuole, and for proteins recycling to the PM (Lam et al., 2007). Traditionally, vacuolar transport from the TGN/EE to PVC/MVB is assumed to be mediated by clathrin-coated vesicles (CCVs). However, recent data reveal that the plant PVC/MVBs are derived from the tubular–vesicular TGN/EE through a process of maturation (Scheuring et al., 2011). The presence of ILVs in the PVC/MVB is formed by the invaginations of limiting membrane of the PVC/MVB into the lumen. Because of the formation of ILVs, the endocytosed membrane proteins transported to the PVC/MVBs can further be separated into two groups: one group of membrane proteins is located on the outer membrane of the PVC/MVBs, while the other group is further sorted into ILVs of the PVC/MVB. Such a separation is critical to ensure that the two types of cargoes either are transported into the vacuole lumen for degradation or remain on the tonoplast after the PVC/MVB–vacuole fusion or are integrated into retrograde transport vesicles (Huotari and Helenius, 2011).

It has been demonstrated that the formation of ILVs in the PVC/MVBs and the sorting of ubiquitinated membrane proteins require endosomal sorting complex required for transport (ESCRT) machinery. In mammalian cells and yeast, the ESCRT machinery consists of five distinct ESCRT complexes (ESCRT-0, -I, -II, -III, and the Vps4 complex) and several ESCRT-associated proteins (Saksena et al., 2007; Williams and Urbé, 2007; Hurley, 2008). Current models from these organisms indicate that the ESCRT-0 complex initially recognizes and clusters the ubiquitinated cargoes and then is recruited to the endosomal membrane by interaction with phosphatidylinositol 3-phosphate (PI3P). ESCRT-I and ESCRT-II are then sequentially recruited by ESCRT-0 to the membrane, which, in turn, passes the cargoes onto the ESCRT-III complex. Then, the cargoes are deubiquitinated and the ILVs are cleaved by ESCRT-III from the endosomal membrane and further dismantled by the VPS4 complex. In plant genome, most ESCRT isoforms have been identified and their interaction networks are largely conserved, except for the canonical ESCRT-0 subunits and the ESCRT-I subunit MVB12 (Figure 2B; Richardson et al., 2011; Shahriari et al., 2011). Interestingly, plants have evolved unique ESCRT components to regulate ESCRT-mediated processes, such as the VPS4/SKD1 ATPase positive regulator POSITIVE REGULATOR OF SKD1 (PROS), the TOM1-LIKE (TOL) family proteins, and the ESCRT-0 function like protein FYVE DOMAIN PROTEIN REQUIRED FOR ENDOSOMAL SORTING 1 (FREE1) in Arabidopsis (Korbei et al., 2013; Gao et al., 2014; Reyes et al., 2014). Moreover, using genetic suppressor screening of *FREE1* mutant, a plant-specific BRO1-DOMAIN PROTEIN AS FREE1 SUPPRESSOR (BRAF) and the RESURRECTION1 (RST1) have been identified recently (Shen et al., 2018; Zhao et al., 2019). BRAF may function as a negative regulator of ESCRT in plant. Additionally, the suppressor protein RST1 identified a FREE1-independent backup pathway that may mediate when needed, which supports the previous finding that the ILVs are still formed in the lumen of the MVBs despite the fact that all four ESCRT complexes are silenced, and thus indicating the presence of ESCRT-independent mechanisms of MVB biogenesis (Theos et al., 2006). Although multiple plant unique ESCRT components and regulators have been recovered recently, several important questions on which protein(s) fulfill ESCRT-0 function in plant and from where initial recognition of ubiquitinated cargo for sorting remain largely unclear.

Distinct from mammalian and yeast cells where most of the ESCRT localize at the MVBs membrane, the plant ESCRT subunits have differential distribution along the endosomal sorting route. The plant ESCRT component TOL6 shows both PM and TGN localization patterns under a confocal microscope (Korbei et al., 2013). In an immuno-EM labeling study, endogenous ESCRT-I subunit VPS28 mainly localizes to the Golgi apparatus and the TGN, rather than to the PVC/MVBs (Scheuring et al., 2011). Moreover, the ESCRT-II subunit VPS22 mainly localizes to TGN, whereas the ESCRT-III subunit VPS2 localizes principally to subdomains of MVBs, and either adjacent to or partially to TGN (Scheuring et al., 2011; Cai et al., 2014). Thus, the different distribution patterns of plant ESCRT components suggest that ESCRT sorting may occur at PM, and the PVC/MVBs start maturing from the specific subdomain of the EE/TGN, which supports the ultrastructure EM observation that PVC/MVBs mature from the tubular-vesicular TGN/EE (Scheuring et al., 2011).

## PVC/MVBS and Vacuole Membrane Interaction: Vacuolar Protein Delivery

The fusion of the PVC/MVBs with the vacuole is the final delivery step for soluble cargoes and membrane proteins into vacuole. This process can be divided into three sequential steps: organelle tethering, *trans*-SNARE complex formation, and membrane fusion. Consistently, the identified regulators that play a role in the PVC/MVBs with the vacuole fusion steps are very conserved in eukaryotic cells. However, distinct from yeast and animal cells, plant cells contain two functional and morphological distinctive forms of vacuoles: LVs and PSVs. Thus, plants probably have evolved a unique mechanism in endomembrane trafficking to maintain development and survival under various stress conditions (Yang and Guo, 2018). The plant LVs contain acid hydrolases playing an important role in multiple biological processes such as protein turnover, abiotic, and biotic stresses defense, and keeping cellular homeostasis (Tan et al., 2019), whereas the PSVs mainly function to store proteins (Jiang et al., 2001). Interestingly, PSVs can convert into LVs during seed germination while LVs can also be replaced by PSVs in leaf cells, although the detailed mechanism of the LV-PSV transition is still largely unknown (Jiang et al., 2001; Feeney et al., 2018; Kwon et al., 2018). The soluble proteins in LVs are mainly transported by the PVC/MVB-mediated vacuolar pathway, while the storage proteins in PSVs are transported by diverse routes. The fusion of PVC/MVBs with PSVs can mediate delivery of proteases to PSVs in protein mobilization in germinating mung bean seeds (Wang et al., 2007; Reyes et al., 2011). In addition, proteins located in plant unique PSVs can also be transported via CCVs, dense vesicles (DVs) (Hohl et al., 1996; Hara-Nishimura et al., 1998), precursor-accumulating vesicles (PACs), ER-derived dark intrinsic protein (DIP) (Jiang et al., 2000), and protein bodies (PBs) (Levanony et al., 1992; Rubin et al., 1992).

To identify the protein machinery that is involved in proteins targeting LVs or PSVs, several unique genetic screening assays have been raised in plant: (1) screening for *maigo* (*mag*) mutants that have accumulated 12S globulin and 2S albumin based on the many novel structures in dry seed under electro-microscope (Shimada et al., 2006; Takahashi et al., 2010; Li et al., 2013); (2) screening *green fluorescence seed* (*gfs*) mutants that have a defect in vacuolar sorting of GFP-CT2, based on the detection of fluorescent signals in the apoplasm (Shimada et al., 2003; Fuji et al., 2007; Tamura et al., 2007); and (3) screening *modified transport to the vacuole* (*mtv*) mutant, based on the interfered secretion of a vacuolar marker (VAC2) to the apoplasm that finally causes the early termination of meristems (Sanmartín et al., 2007). By this way, several regulatory proteins have been isolated and identified, which are required for PVC/MVB-mediated proteins in vacuolar targeting. For example, the MAG1 protein, which encodes the core retromer component VPS29, may be involved in retrograde trafficking of membrane proteins (e.g., VSRs and PINs) from the PVC/MVBs to the TGN or other unclarified endosomes (Figure 2C; Shimada et al., 2006); the AP-4 protein, one subunit of adaptor complex protein, encoded by GFS4 or GFS5, interacts with VSR1 at TGN to participate in vacuolar protein sorting (Fuji et al., 2016); MTV1 and MTV4, which encode an epsin N-terminal homology (ENTH) protein and an ADP ribosylation factor (ARF) GTPase-activating protein (GAP) AGD5, respectively, are functioning in mediating clathrin-dependent targeting of vacuolar cargoes from the TGN to the PVC/MVBs (Sauer et al., 2013). Moreover, VSR4 encoded by MTV2 has also been elucidated to participate in the regulation of vacuolar protein sorting (Zouhar et al., 2010).

**FIGURE 3:**
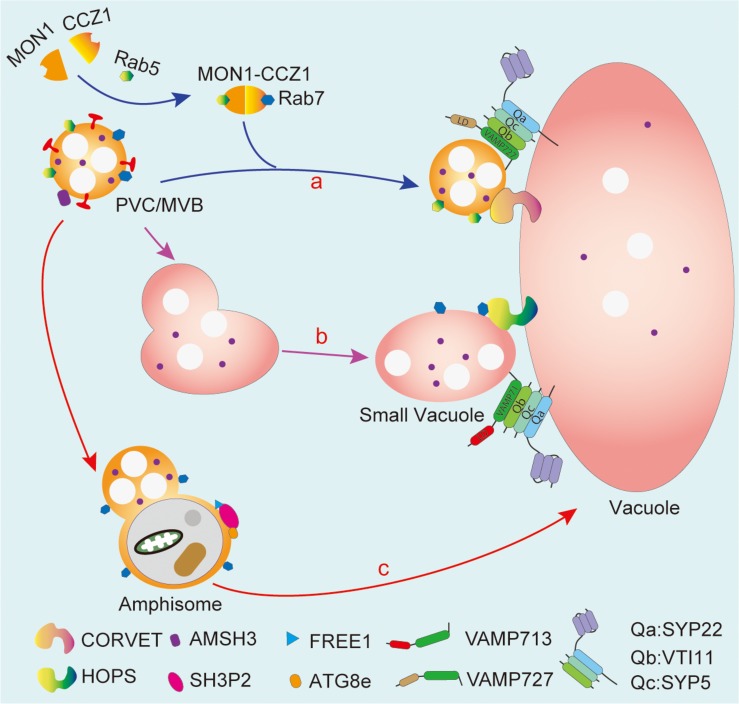
PVC/MVBs mediated vacuolar transport and crosstalk with autophagosome. PVC/MVB maturation requires the MON1 and CCZ1 complex-mediated conversion between Rab5 and Rab7. The CORVET complex coordinated with RAB5 and the VAMP727-containing SNARE complex bridge the fusion between PVC/MVBs and the vacuole (route **a**). On the other hand, PVC/MVBs can heterotypically fuse with the small vacuole and then fuse with the central vacuole, which is mediated by HOPS and the VAMP713-containing SNARE complex (route **b**). The crosstalk between PVC/MVBs and autophagosome aims to form amphisome that may deliver cargo into the vacuole via the fusion between the amphisome and the vacuole (route **c**). MON1, monensin sensitivity 1; CCZ1, calcium caffeine zinc sensitivity 1; CORVET, class C core vacuole/endosome tethering; HOPS, homotypic fusion and protein sorting. SH3P2, SH3 domain-containing protein 2; VAMP, vesicle-associated membrane protein; VTI11, vesicle transport v-SNARE 11; SYP, syntaxin of plants.

Currently, by combining genetic tools and molecular cell biology approaches, the mechanism as to how PVC/MVBs deliver soluble proteins to the LV/PSVs has been extensively explored (Figure 3). For instance, the Rab7 family proteins, the guanine nucleotide exchange factor VPS9a, and the MON1-CCZ1 (MONENSIN SENSITIVITY1-CALCIUM CAFFEINE ZINC SENSITIVITY1) protein complex have been proven to be critical for the PVC/MVB-mediated vacuolar protein trafficking in Arabidopsis (Cui et al., 2014; Ebine et al., 2014; Singh et al., 2014). Besides, Q-SNARE VTI11/12 and R-SNARE VAMP727 have also been demonstrated to regulate the formation of PSVs from PVC/MVBs (Sanmartín et al., 2007; Ebine et al., 2008). In addition, two evolutionarily conserved tethering complexes, class C core vacuole/endosome tethering (CORVET) and homotypic fusion and protein sorting (HOPS), are proposed to be involved in mediating membrane fusion process in vacuolar targeting pathways by coordination with different sets of SNARE proteins and RAB GTPase. For more details, CORVET coordinates with RAB5 and the VAMP727-containing SNARE complex to mediate membrane fusion between PVC/MVBs and the vacuole (Figure 3, route a, blue arrow), while HOPS functions together with RAB7 and the VAMP713-containing SNARE complex to regulate membrane fusion between small vacuoles (Hao et al., 2016; Brillada et al., 2018; Takemoto et al., 2018; Figure 3, route b, purple arrow).

Interestingly, PVC/MVBs not only play an important role for proteins in vacuole sorting but also are required for the formation of central vacuole. Recent 3D electron tomography investigations have demonstrated that small vacuoles served as nascent vacuoles in LV biogenesis and parts of small vacuoles are originally derived from PCV/MVBs *via* heterotypic fusion between the PVC/MVBs and small vacuoles (Cui et al., 2019). Moreover, the SNARE protein VTI11 and the newly identified ESCRT component FREE1 are essential for both the formation of small vacuoles and the heterotypic fusion of small vacuoles with the PVC/MVBs. Importantly, the ESCRT component AMSH3 (Isono et al., 2010) and FREE1 (Gao et al., 2015) have also been found to regulate the vacuolar transportation of storage proteins, although the molecular base of the underlying mechanism is still largely unknown. In addition to the function of FREE1 in endosomal trafficking, a recent study demonstrates that FREE1 has a crosstalk with ABA signal pathway (Belda-Palazon et al., 2016; Li et al., 2019). Consistently, it is proposed that the VSR-mediated vacuolar protein targeting is also required for ABA biosynthesis induced by osmotic stress of plants (Wang Z. -Y. et al., 2015). Thus, it will be interesting in the future to clarify the crosslink between vacuolar protein trafficking machinery and behaviors of phytohormone that balance the plant growth and stress response.

## PVC/MVBS and Autophagosome Membrane Interactions: Crosstalk With the Autophagy Pathway

Macroautophagy (hereafter autophagy) is an evolutionarily conserved self-eating process in eukaryotes by forming a double-membrane-bound autophagosome, to engulf a portion of cytoplasmic materials or damaged organelles into lysosome/vacuole for degradation. Autophagosome formation starts from phagophore, followed by the expansion and closure of the double membrane to form a globular double-membrane structure (Mizushima et al., 2011; Zhang and Zhang, 2016). It has been suggested that ER is the major membrane origin of autophagosomes (Hayashi-Nishino et al., 2009; Ylä-Anttila et al., 2009). However, increasing evidences have also been demonstrated for the coordination and crosstalk between autophagosome and the PVC/MVB pathway (Figure 1, route f, red solid arrow), as well as for dual roles of the PVC/MVB localized proteins functioning in vacuolar transport and autophagosome formation (Figure 3, route c, red solid arrow; Kulich et al., 2013; Gao et al., 2015; Cui et al., 2018).

The crosstalk between the PVC/MVBs and autophagosomes may contribute to the complex network of plant stress responses (Wang et al., 2020). The first reported piece of evidence in Arabidopsis is the PVC/MVB-localized ESCRT-III-associated deubiquitinating enzyme Associated Molecular with SH3 domain of STAM 3 (AMSH3). Mutation of *amsh3* fails to form the central vacuole and causes a large number of autophagosome accumulation, which indicates a malfunction of autophagic degradation (Isono et al., 2010; Katsiarimpa et al., 2011). In addition, the same subcellular phenotypes can also be found in the AMSH3-related deubiquitinating enzyme, *amsh1*-knockdown mutant, and its interaction partner, the ESCRT-III components *vps2.1* mutant. Impairment of AMSH1 or VPS2.1 causes a hypersensitivity response to starvation and early senescence, similar to autophagy related gene mutants. Consistently, the YFP-ATG8e labeled autophagosomes accumulate in the cytoplasm of the mutants but are barely observable in the vacuole lumen, probably because the maturation of autophagosomes is disturbed and results in insufficient trafficking of autophagosomes to the vacuole (Katsiarimpa et al., 2013). Another ESCRT-III protein showing an autophagy-related phenotype is the Arabidopsis Charged Multivesicular Body Protein1 (CHMP1), where the unclosed autophagosomes accumulate in the *chmp1* mutant. Besides, CHMP1 plays a direct role in the autophagosome-mediated degradation of chloroplast proteins into the vacuoles and nutrient recycling under starvation (Spitzer et al., 2015). Moreover, in the mutant of plant unique ESCRT component FREE1, the formation of ILVs is strongly impaired and also results in an abnormal accumulation of autophagosomes and MVB–autophagosome hybrid structures, pointing to a dual role for ESCRT components in regulating vacuolar protein transport and the autophagic degradation pathway (Hurley and Hanson, 2010; Zhuang et al., 2013; Gao et al., 2014, 2015; Cardona-López et al., 2015; Shen et al., 2016). Interestingly, FREE1 can directly interact with SH3 DOMAIN-CONTAINING PROTEIN2 (SH3P2), which is also found to be specifically translocated to the PAS upon autophagy induction and contributes to membrane deformation in cooperation with the phosphatidylinositol 3-phosphate kinase (PI3K) complex (Zhuang et al., 2013; Gao et al., 2015). In addition, it has been elucidated that SH3P2 could interact with the ESCRT component Vps23 and AMSH3 at CCVs, thus proposing that SH3P2 may also function to recognize the ubiquitinated membrane proteins and deliver them to the ESCRT machinery in clathrin-mediated endocytosis (Nagel et al., 2017). Considering both SH3P2 and FREE1 have dual functions in ESCRT-dependent vacuolar trafficking pathway and autophagy, the endocytic pathway mediated by the PVC/MVBs may operate critical role(s) in autophagosome formation and maturation.

In addition to the ESCRT proteins, the Rab7 family proteins may be involved in the autophagosome pathway. It is reported that RABG3b, a homolog of Rab7 GTPase, colocalizes with ATG8a-labeled autophagosome in an immunogold transmission electron microscope (TEM) study under pathogen infection. Interestingly, overexpression of the constitutively active form RABG3b (RABG3bCA) could restore autophagic activity in the *atg5-1* mutant. By contrast, the programed cell death is accelerated in overexpressed RABG3bCA transgenic plant upon *P. syringae* treatment (Kwon et al., 2010, 2013). However, more reliable evidences identifying regulators to support the membrane interaction between the endocytic PVC/MVBs and the autophagosome are still in high demand. To achieve this, it can be especially helpful to explore the molecular mechanisms by learning the PVC/MVB–autophagosome crosstalk employed in mammals and yeast cells.

Consistent with that in plants, it has been reported that several components of the ESCRT machinery have a crosstalk in both autophagic and MVB-mediated lysosome trafficking pathways in mammalian cell (Lamb et al., 2013). For example, mutation of the hepatocyte growth factor-regulated tyrosine kinase substrate (HRS) compromised the degradation ability of aggregated proteins in autophagic pathway and caused enhanced ER stress (Oshima et al., 2016), while dysfunction of the TOM1 in mammals inhibits the fusion between autophagosome and lysosome that blocked the autolysosome formation (Tumbarello et al., 2012). Both the HRS and TOM1 are ESCRT-0 components in mammals, thus indicating that ESCRT-0 is required for the proper function of autophagosome. Another ESCRT component involved in autophagosome biogenesis is mammalian ALG-2 interacting protein X (Alix), which can interact with the ATG2–ATG3 complex to accelerate basal autophagic flux (Petiot et al., 2008; Murrow et al., 2015). Besides the ESCRT machinery, Rab GTPase family proteins also have been elucidated to participate in EE and autophagosome formation. Inhibition of Rab5 activity not only inhibits endosome maturation but also decreased the number of autophagic bodies in mammals (Zeigerer et al., 2012). Similarly, Rab7 GTPases play multiple roles in the LEs/MVBs and autophagosome maturation processes, as well as in their fusion with the lysosome in mammalian cells (Hyttinen et al., 2013; Singh et al., 2014). So far, reports on crosstalk between MVB and the autophagic pathway remain lacking, probably because most of the MVB-localized proteins are functionally essential, and their mutation may lead to cell death. Future studies using better genetic screening system (e.g., conditional induced RNAi system) would help us to identify and characterize new regulators that are involved in the MVB and autophagosome crosstalk pathways.

## PVC/MVBS and Plasma Membrane Interactions: Unconventional Secretion

In the classical or conventional protein secretion pathway, secretory proteins lacking a vacuolar sorting signal can transport through the TGN/EE and then move into the extracellular space (ECS) (Figure 1, route b, purple dashed arrow). Interestingly, it has now been demonstrated that plants also make use of different types of unconventional protein secretion pathways (Drakakaki and Dandekar, 2013): a Golgi–bypass secretion pathway for signal peptide-lacking cytosolic proteins (Cheng et al., 2009; Zhang et al., 2011) and secretion pathways mediated by specific organelles including the central vacuole (Hatsugai et al., 2009), the PVC/MVBs (Wang et al., 2011; Nielsen et al., 2012; Nielsen and Thordal-Christensen, 2013), and a double-membrane organelle termed exocyst-positive organelle (EXPO). Recent findings suggest that the inner membrane of EXPO can be released to ECS as an exosome-like structure upon EXPO–PM fusion (Wang et al., 2010), although EXPO may also be related with autophagy (Lin et al., 2015; Peěenková et al., 2018).

In mammals, exosome is the name given to vesicles that are released through the fusion of these specific organelles with the PM. Generally, exosomes have a diameter between 50 and 150 nm and appear to be involved in the transport of numerous proteins, lipids, sRNA, and chemicals into the extracellular spaces enabling cell-to-cell communication and defense response (Théry et al., 2002; Valadi et al., 2007; Kowal et al., 2014; Tao et al., 2019). The ILVs inside the MVBs are the major source of exosomes that are released into ECS upon the MVB–PM fusion (Colombo et al., 2014). Currently, several components have been identified as being involved in the regulation of the PVC/MVB–PM fusion process, including the cytoskeleton, Rab GTPases, and SNAREs in yeast and mammals (Granger et al., 2014; Mathieu et al., 2019). Because cytoskeleton is required for the movement of MVBs to PM (Granger et al., 2014; Sinha et al., 2016), the exosome release event is substantially inhibited or promoted during knockdown or overexpression of the actin binding protein cortactin. In cortactin knockdown cells, both the number of motile MVBs and the number of PM docking sites are decreased, whereas overexpression of cortactin increases the efficiency of MVB–PM docking (Sinha et al., 2016). Likewise, Rab GTPases have also been identified as regulators in the exosome secretion process, although the precise mechanism is not well understood. For example, overexpression of a dominant-negative form or mutation of Rab11 in human leukemic K562 or *Drosophila* S2 cells causes inhibition of exosome release and a reduction in the number of exosomes (Savina et al., 2002). Disruption of Rab35 causes impaired secretion of proteolipid protein (PLP)-bearing exosomes in Oli-neu cells, which may be due to the blockage of the MVB–PM docking (Hsu et al., 2010). More strikingly, the morphology of MVBs is abnormal and the ability of MVBs tethering to the PM is reduced in Rab27a or Rab27b deficiency mutants (Ostrowski et al., 2010). In addition to Rab family proteins, SNARE proteins are also required for both MVB docking and fusion with PM. When the N-terminal domain of the R-SNARE vesicle-associated membrane protein 7 (VAMP7) is overexpressed, the formation of the specific SNARE complex and exosome release are blocked due to the accumulation of enlarged MVBs at cell periphery, indicating that the correct expression of VAMP7 is critical for MVB–PM fusion and exosome release in the human cell line K562 (Fader et al., 2009). Consistently, another R-SNARE protein that may participate in MVB–PM fusion is YKT6, which is essential for Wnt-bearing exosome secretion in mammalian cells (Gross et al., 2012). Moreover, the synaptosomal-associated protein 23 (SNAP-23), a plasma membrane-associated SNARE protein, has also been revealed to play a function in MVB–PM fusion in HeLa cells (Verweij et al., 2018).

**FIGURE 4:**
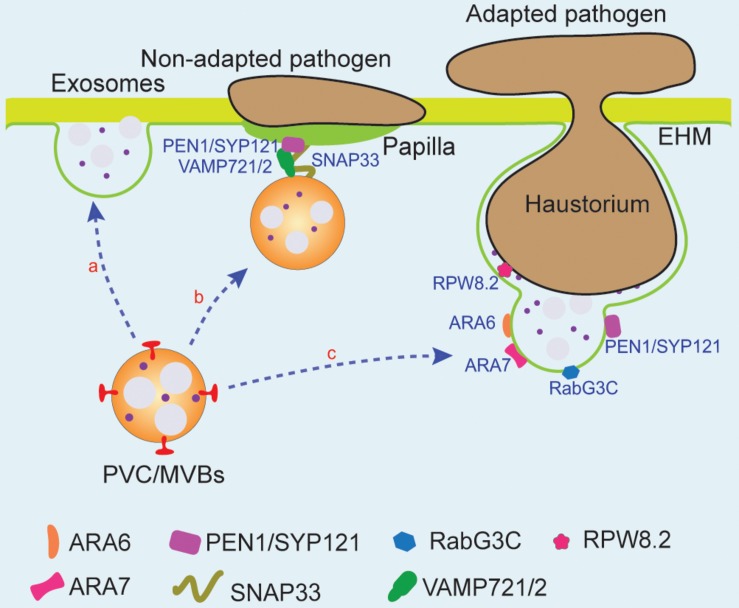
PVC/MVBs and PM membrane fusion is involved in pathogen response. PVC/MVBs can fuse with PM to form exosomes (route **a**). PVC/MVBs accumulated at the non-adapted pathogen infection site to secrete materials and proteins, which are required for the formation of papilla. Three PM-associated SNARES, PEN1/SYP121, VAMP721/2, and SNAP33, can be found at the infection site of powdery mildew (route **b**). Upon the adapted fungal pathogen penetration, Arabidopsis disease resistance protein, RPW8.2, can be recruited to the EHM and function as the host border control mechanism of plant–pathogen interaction. PVC/MVBs that accumulate at the periphery of PM-haustorium and fuse with EHM may participate in the formation and modulation of the EHM with the assistance of several regulators such as ARA6, ARA7, and Rab7 GTPase and thus release resistance-related proteins (route **c**). EHM, extrahaustorial membrane; PEN1, penetration 1; RPW8.2, resistance to powdery mildew 8.2; SNAP33, SNAP25 homologous protein SNAP33; SNARES, soluble NSF attachment protein receptors.

As in mammalian cells, exosomes in plant cells are mainly derived from the membrane fusion of the PVC/MVBs with PM (An et al., 2007; Rutter and Innes, 2017; Figure 4, route a). The function of exosomes in the plant extracellular space is diverse (de la Canal and Pinedo, 2018). Most importantly, it has been suggested that the release of exosomes may be involved in plant–pathogen interactions (Hansen and Nielsen, 2017; Li et al., 2018). Numerous membrane-bound vesicle-like structures with diameters of 60–150 nm that accumulated in the extracellular space of tobacco leaf tissue can be observed under turnip mosaic virus (TuMV) invasion conditions, and these structures appear to be ILVs (Movahed et al., 2019). Similarly, upon the attachment and germination of non-adapted fungal spores on leaves, the secretion of exosomes is induced, which contains numerous molecules that form a type of cell wall apposition or papilla followed by the fusion of the PVC/MVBs with the PM (An et al., 2006). Besides the materials for the formation of papilla, exosomes are also involved in transporting small RNAs (sRNAs), which could be taken up by fungal cells to silence virulence effectors secreted by pathogens (Cai et al., 2018). Indeed, upon infection with the bacterial pathogen *Pseudomonas syringae*, the activity of exosome secretion is substantially enhanced. Proteomic analyses of the exosomes, extracted from *P. syringae* or salicylic acid (SA)-treated Arabidopsis rosettes, have demonstrated that exosomes contribute to the release of pathogen resistance-related compounds, including RPM1-INTERACTING PROTEIN 4 (RIN4), RIN4-interacting proteins, and PENETRATION1 (PEN1) (Rutter and Innes, 2017).

PEN1 is a PM-syntaxin, syntaxin of plants (SYP)121, which binds with other SNAR proteins to form PM-localized ternary SNARE complexes to resist non-adapted pathogens (Collins et al., 2003; Kwon et al., 2008). Facing the penetration by *Blumeria graminis* f. sp. *Hordei* (*Bgh*), an adapted pathogen of barley and non-adapted pathogen of Arabidopsis, PEN1 timely interacts with SNAP33, VAMP721/722, and ARF GTPase to accelerate the accumulation of exosomes at infection sites to form papillae, which is essential for cell wall-based defense of non-adapted pathogens (Assaad et al., 2004; Nielsen et al., 2012). In addition, evidences have shown that the GFP-PEN1 can be found outside the PM and colocalized with the FM4-64-labeled structures during non-adapted pathogen infection; thus, the distribution of the PEN1/SYP121 is indeed altered from PM to exosomes. GFP-PEN1 can also be detected at the extracellular matrix region in the penetration site and the extrahaustorial membrane of Arabidopsis adapted powdery mildew pathogen *Golovinomyces cichoracearum* (Nielsen et al., 2012). More interestingly, the number of ARA6-GFP-labeled PVC/MVBs also increased and accumulated at the attack site under both fungal and bacterial pathogen infection conditions, indicating that GFP-PEN1-positive membrane material sorting to papillae at the infection site may be the result of fusion between PVC/MVBs and PM (Meyer et al., 2009; Nielsen et al., 2012; Nielsen and Thordal-Christensen, 2013; Figure 4, route b). The fungal pathogen PEN1 is also found enriched in extracellular vesicles and papillae in the infected leaf cells upon bacterial pathogen infection and SA treatment (Assaad et al., 2004; Rutter and Innes, 2017; Li et al., 2018). Interestingly, upon barley powdery mildew fungus invasion, the syntaxin-associated VAMP721/722 can also mediate plant phospholipase PLDδ secretion at penetration sites, function as a regulatory mechanism in plant innate immunity (Xing et al., 2019). Another SNARE protein that may be involved in plant–pathogen interactions is Qc-SNARE BET12, which functions in the protein secretion, because its ectopic expression affects pathogenesis-related 1 (PR1) secretion to ECS (Peěenková et al., 2017; Chung et al., 2018).

Besides SNARE proteins, Rab GTPases have been identified to participate in the PVC/MVBs and the PM fusion during host–microbe interfaces (Collins et al., 2003; Bozkurt et al., 2015). Rab5 GTPase has two type homologs, the plant unique ARA6 and the conventional ARA7, while Rab7 GTPase has eight homologs (Lee et al., 2004; Mackiewicz and Wyroba, 2009). ARA6 localizes at the membrane of the PVC/MVBs and mediates the fusion of the PVC/MVBs with the PM at the infection site of the fungal pathogen *Botrytis cinereal*. After fusion, exosomes are released to ECS, whereas ARA6 remains at PM to regulate the next round of transport of the PVC/MVBs (Inada et al., 2016). For successful penetration, filamentous pathogens can form a special feeding structure into host plant cells, which is called a haustorium. This structure is surrounded by a domain of the PM of the host cell, and thus is also called extrahaustorial membrane (EHM) (Roberts et al., 1993; Figure 4, route c). Another Rab5 GTPase ARA7, known as a marker of the PVC/MVBs, is also found to accumulate at the EHM to regulate resistance-related molecule secretion upon invasion by powdery mildew fungus (Inada et al., 2016). Thus, these results suggest that Rab GTPases are involved in rerouting of the PVC/MVBs to the host–pathogen interfere sites. Interestingly, it is also raised that the re-routed vacuole-targeted PVC/MVBs may function as a membrane source of the EHM, because the PVC/MVBs and tonoplast-localized Rab7 GTPase RabG3c are recruited and redirected to the haustorial interface after infection with the oomycete pathogen *Phytophthora infestans* in tobacco leaves (Lu et al., 2012; Bozkurt et al., 2015). However, direct immuno-EM images showing the origin of EHM from PVC/MVBs is still lacking. Moreover, it is still unknown if any effector secreted by the pathogen can be recognized by the host to trigger the movement of the PVC/MVBs to the EHM (Wang W. et al., 2009; Kim et al., 2014; Berkey et al., 2017). Recently, the Arabidopsis pattern recognition receptor FLS2 and the Arabidopsis resistance to powdery mildew8.2 (RPW8.2) have been found to be recruited to the EHM upon pathogen infection and defined as a host border control system that plays a role in the plant–pathogen interface. Thus, it is highly possible that the route of PVC/MVB to PM may depend on certain effector(s) from pathogens that can be recognized by PM-localized receptors, and finally triggers the EHM formation.

In addition, the ESCRT machinery may play a role in the plant–pathogen interaction site by regulating PVC/MVB formation. Indeed, the ESCRT regulatory protein LIP5 is involved in the basal resistance response to the bacterial pathogen *P. syringae*, since the formation of both the PVC/MVBs and the EVs were significantly compromised in *lip5* mutant upon pathogen invasion (Wang et al., 2014; Wang F. et al., 2015). However, the underlying mechanism as to how the pathogen triggers accumulation of the PVC/MVBs at infection sites remains unclear.

Up to now, more specific protein pairs that are supposed to function in the PVC/MVB–PM fusion have been identified using artificial protein fusions or bimolecular fluorescence complementation (BiFC) approach. It is demonstrated that the remorin StRem1.3, receptor-like kinases BIK1, PBS1, CPK21, as well as the PtdIns(4)-binding proteins FAPP1 and Osh2, function as the PM-binding proteins. The Rab5 GTPases (RHA1, ARA6, and ARA7), the Rab7 GTPase RABG3f, and the PtdIns(3)P-binding proteins Vam7p and Hrs-2xFYVE act as tethering proteins on the PVC/MVBs to participate in PVC/MVB–PM tethering. Although various membrane-trafficking proteins have been supposed to function in the PVC/MVB–PM fusion in plants, the precise mechanism that mediates the fusion processes remains largely unknown (Tao et al., 2019).

## Conclusion and Future Perspectives

Over the past decades, substantial progress has been made in establishing the critical roles of PVC/MVBs in endosomal trafficking pathways in plants. Traditionally, the PVC/MVBs play an important role in both secretory pathway as intermediate compartments and endocytic pathway as late endosomes. However, recent discoveries have now revealed that the PVC/MVBs also participate in multiple fusion processes related to autophagy or unconventional secretion.

These diverse functions place the PVC/MVBs on the center stage, but there remain several intriguing questions for future studies. (1) There remain many challenges to achieving a full understanding of the PVC/MVB biogenesis, especially the diverse distribution of the ESCRT machinery components in plants. Moreover, we need better ways to distinguish the PVC/MVBs from Late PVCs (Foresti et al., 2010) and to characterize the molecular steps and processes occurring in the maturation of the PVC/MVBs. (2) In yeast, after MVB–vacuole fusion, many of these proteins and lipids remain in the vacuole membrane while some are degraded in the vacuole lumen, and others may even be recycled (Suzuki and Emr, 2018). It will be important and interesting to address the fate of plant PVC/MVB membrane proteins after fusion with the tonoplast and to determine which key proteins (e.g., SNAREs, receptors) are involved in retrograde vacuole-to-endosome trafficking. (3) There are increasing evidences for the operation of a crosstalk between the PVC/MVB pathway and the autophagy pathway, as well as for dual roles of the PVC/MVB-localized protein function in vacuolar protein transport and autophagosome formation. However, which regulators (such as SNAREs and Rab GTPases) mediate the PVC/MVB–autophagosome fusion process in plants remain to be elucidated. In addition, although MVB–autophagosome hybrid ultrastructures have been proposed in *free1* mutant under TEM, the dynamics of the fusion steps of the MVBs and autophagosome are still missing. Importantly, it is also interesting to know that the MVB–autophagosome fusion is induced in general stress condition(s) or specific conditions. Future studies on autophagy and the PVC/MVB pathways under multiple stresses or nutrient limitation conditions would lead us to a better understanding of their coordination and crosstalk in plants. (4) Further progress still needs to be made in identifying additional components important for pathogen-responsive PVC/MVB biogenesis and associated PVC/MVB–PM fusion. The proteomic profiling of their contents from isolated plant extracellular vesicles and MVB-derived exosomes can further provide fundamental information for the roles of PVC/MVBs in plant–pathogen interactions (Heard et al., 2015; Rutter and Innes, 2017). Potential regulatory activities in cell-to-cell communication or in plant–pathogen interactions also need to be investigated. Although pioneering studies have shed light on the roles of trafficking related proteins in PVC/MVB–PM fusion steps, future studies using high-resolution 3D TEM prepared from high-pressure freezing/freeze substitution fixation (HPF/FS) are still considered useful tools to reveal fusion steps and the underlying mechanism of this progression.

Compared to the tremendously diverse roles of the MVBs in mammals and yeast, the reported functions of the PVC/MVBs in plants remain somewhat limited. State-of-the-art microscopy technology, such as super-resolution fluorescence microscopy with 3D structures in living cells, light-sheet microscopy for 4D imaging, as well as 3D TEM and correlative light-electron microscopy (CLEM) approaches (Feeney et al., 2018; Wang et al., 2019), can be expected to be useful tools for the analysis of endosomal structure and trafficking when plants are placed under multiple environments. This will undoubtedly bring new insights into our understanding of the PVC/MVB membrane interaction network and their associated functions.

## Author Contributions

JS conceived the idea. JS and SH wrote the manuscript. JS, SH, and YL evaluated the manuscript. All authors read and approved the manuscript.

## Conflict of Interest

The authors declare that the research was conducted in the absence of any commercial or financial relationships that could be construed as a potential conflict of interest.
